# Deregulation of lysophosphatidic acid metabolism in oral cancer promotes cell migration via the up-regulation of COX-2

**DOI:** 10.7717/peerj.10328

**Published:** 2020-11-11

**Authors:** Mariati Abdul Rahman, May Leng Tan, Steven P. Johnson, Robert J. Hollows, Wen Lin Chai, Jason P. Mansell, Lee Fah Yap, Ian C. Paterson

**Affiliations:** 1 Department of Oral and Craniofacial Sciences, University of Malaya, Kuala Lumpur, Malaysia; 2 Department of Craniofacial Diagnostics and Biosciences, Universiti Kebangsaan Malaysia, Kuala Lumpur, Malaysia; 3 North Devon Healthcare NHS Trust, Barnstable, United Kingdom; 4 Institute of Immunology and Immunotherapy, University of Birmingham, Birmingham, United Kingdom; 5 Department of Restorative Dentistry, University of Malaya, Kuala Lumpur, Malaysia; 6 Department of Applied Sciences, University of the West of England, Bristol, United Kingdom; 7 Oral Cancer Research and Coordinating Centre, University of Malaya, Kuala Lumpur, Malaysia

**Keywords:** LPA, OSCC, Migration, Invasion, Radio-resistance, COX-2

## Abstract

Oral squamous cell carcinoma (OSCC) is the sixth most common cancer worldwide and accounts for 300,000 new cases yearly. The five-year survival rate is approximately 50% and the major challenges to improving patient prognosis include late presentation, treatment resistance, second primary tumours and the lack of targeted therapies. Therefore, there is a compelling need to develop novel therapeutic strategies. In this study, we have examined the effect of lysophosphatidic acid (LPA) on OSCC cell migration, invasion and response to radiation, and investigated the contribution of cyclooxygenase-2 (COX-2) in mediating the tumour promoting effects of LPA. Using the TCGA data set, we show that the expression of the lipid phosphate phosphatases (LPP), LPP1 and LPP3, was significantly down-regulated in OSCC tissues. There was no significant difference in the expression of the ENPP2 gene, which encodes for the enzyme autotaxin (ATX) that produces LPA, between OSCCs and control tissues but ENPP2 levels were elevated in a subgroup of OSCCs. To explore the phenotypic effects of LPA, we treated OSCC cell lines with LPA and showed that the lipid enhanced migration and invasion as well as suppressed the response of the cells to irradiation. We also show that LPA increased COX-2 mRNA and protein levels in OSCC cell lines and inhibition of COX-2 activity with the COX-2 inhibitor, NS398, attenuated LPA-induced OSCC cell migration. Collectively, our data show for the first time that COX-2 mediates some of the pro-tumorigenic effects of LPA in OSCC and identifies the ATX-LPP-LPA-COX-2 pathway as a potential therapeutic target for this disease.

## Introduction

Oral squamous cell carcinoma (OSCC) remains a major world health issue and is particularly prevalent in India and Southeast Asia with more than 300,000 new cases being diagnosed each year (Bray et al., 2018). The prognosis of patients diagnosed with OSCC is poor; approximately 50% of patients die within 5 years due to loco-regional recurrence, distant metastases and second primary tumours (Omar, 2013). Whilst our understanding of the molecular basis for the development of OSCC is now relatively extensive (Leemans, Snijders & Brakenhoff, 2018), molecular targeted therapies and novel approaches to treatment are still required.

Lysophosphatidic acid (LPA) is a small glycerophospholipid which is found in all eukaryotic tissues and also in saliva. LPA functions as a bioactive lipid mediator with a variety of biological actions, such as the promotion of cell proliferation, migration and survival (Moolenaar, 2000; Van Corven et al., 1989). LPA is synthesized from its precursor, lysophosphatidylcholine (LPC), by the lysophospholipase D activity of the enzyme, autotaxin (ATX) (Aoki, 2004). Six cell surface G protein-coupled receptors (GPCR; LPAR1-6) have been identified as high affinity cognate LPAR receptors and these receptors are coupled to and activate one or more G protein (G*α*i/0, G*α*q/11, Gs and G*α*12/13) which then stimulate multiple signalling cascades (Yung, Stoddard & Chun, 2014). There is increasing evidence to show that LPA and its receptors play important roles in cancer (Tigyi et al., 2019). For example, LPA has been shown to enhance the migration and invasion in a variety of cancer types including colon, pancreatic and ovarian carcinomas (Kato et al., 2012; Shida et al., 2003; Yu et al., 2008) via the activation of several LPARs. Indeed, aberrant or elevated expression of LPA receptors has been reported in several human malignancies in vivo and in vitro. (Hayashi et al., 2012; Willier, Butt & Grunewald, 2013). Furthermore, ATX/LPA signalling is known to promote resistance to radiotherapy (Brindley, Lin & Tigyi, 2013), possibly by decreasing the proapoptotic effect of ceramides following the activation of PLD and PI3K, which increases the levels of the survival factor sphingosine 1-phosphate (Brindley, Lin & Tigyi, 2013). Extracellular LPA is hydrolysed by three plasma membrane bound lipid phosphate phosphatases, LPP1, LPP2 and LPP3 (encoded by the genes *PPAP2A*, *PPAP2C* and *PPAP2C*, respectively), which attenuates downstream signalling, although it is important to note that intracellular functions of LPPs have been described (Tang & Brindley, 2020)

COX-2 is a membrane bound enzyme which catalyses the generation of prostanoids, such as prostaglandin E2 (PGE2) (Claria, 2003). Overexpression of COX-2 has been detected in various types of cancer with a number of studies reporting that COX-2 expression levels were significantly higher in OSCCs compared to normal tissue (Byatnal et al., 2015; Goulart Filho et al., 2009; Lee, Kang & Kim, 2015; Moazeni-Roodi et al., 2017; Tang et al., 2003) and overexpression of COX-2 correlated with poor prognosis in cervical SCCs following radiation (Gaffney et al., 2001). In OSCC cells, COX-2 has been shown to mediate *α*v*β*6 integrin-dependent invasion (Nystrom et al., 2006) and is associated with radioresistance (Terakado et al., 2004). Interestingly, there is evidence to show that COX-2 can mediate some of the tumour promoting effects of LPA because in ovarian carcinoma cells, LPA-induced migration was attenuated following treatment with NS-398, a COX-2 inhibitor (Symowicz et al., 2005) and the LPAR2-Gi/Src pathway promoted cell motility via COX-2 expression (Jeong et al., 2008).

A recent report suggested that LPA metabolism might be deregulated in head and neck cancers via the downregulation of LPP3 (Tang & Brindley, 2020). In the present study, we have extended these observations and examined the expression of the genes that encode ATX (*ENPP2*), LPP1 (*PPAP2A*), LPP2 (*PPAP2C*) and LPP3 (*PPAP2B*) in OSCC tissues. We also determined the effect of LPA on OSCC-derived cell lines and examined whether these effects are mediated by the up-regulation of COX-2. We show that whilst only a small proportion of OSCCs expressed high levels of *ENPP2*, the expression of both *PPAP2A* and *PPAP2B* was significantly down-regulated in OSCC tissues compared normal controls. Further, LPA promoted cell migration and invasion, and protected OSCC cells from radiation. We further show that LPA up-regulated COX-2 mRNA and protein expression in OSCC cell lines and that inhibition of COX-2 activity attenuated the pro-migratory effects of LPA. Taken together, our data show that LPA metabolism is deregulated in OSCCs and that LPA enhances tumour cell migration by up-regulating COX-2 in OSCC. These data suggest that that the LPPs are potential therapeutic targets for OSCC and that targeting LPA metabolism alongside the inhibition of COX-2 might be useful clinically.

## Materials & Methods

### Materials

18:1 LPA was purchased from Avanti Polar Lipids (Alabaster, AL, USA) and was dissolved in ethanol:water (1:1, v/v) to create a 10 mM stock solution. Stock solutions were stored at −20 °C. NS 398 and Ki 16425 was purchased from Tocris Bioscience (Bristol, UK) and R&D Systems (Minneapolis, USA), respectively.

### Cell lines

Details of the OSCC cell lines have been described previously (Edington et al., 1995; Munro et al., 2001; Prime et al., 1990). Cells were obtained from The European Collection of Authenticated Cell Cultures (ECACC) and were maintained in DMEM/F12 supplemented with 10% (v/v) foetal bovine serum. All cell culture reagents and media were obtained from Gibco (Thermo Fisher Scientific Inc, MA, USA).

### Analysis of *ENPP2* and *PPAP2A-C* expression in The Cancer Genome Atlas HNSCC data set

HNSCC data from The Cancer Genome Atlas (TCGA) were obtained from the TCGA data portal (The Cancer Genome Atlas Network, 2015). Level 3 RNA-sequencing and clinical data were downloaded in the “Biotab” format”. OSCC samples were identified using the “anatomic neoplasm subdivision” field and classified as as either human papillomavirus (HPV)-negative or HPV-positive. In total RNA-seq data were available for 265 HPV-negative OSCC samples, and for 26 of these data were also available for matched “normal” samples.

The edgeR package (Robinson, McCarthy & Smyth, 2010) in R (R Core Team, 2018) was used to normalize read counts between samples and to convert reads for each gene to counts-per-million (cpm). Differential expression analysis was also performed using edgeR and survival analyses were performed using the “survival” package in R.

### Scatter assays

Cell scattering in response to LPA was measured exactly as described previously (Patmanathan et al., 2016). 5 µM epidermal growth factor (EGF) was used as a positive control.

### Transwell migration and invasion assays

For cell migration, polycarbonate filters (8 µM pore size; Transwell, Corning, USA) were coated with fibronectin (10 µg/mL) in 24-well plates (Costar, Corning, USA) for two hours at 37 °C. Cells were cultured in reduced serum (DMEM/F12/1% FBS) overnight and treated with 10 µg/mL mitomycin C for two hours to inhibit cell proliferation. 5 × 10^5^ cells in media with reduced serum containing 0.25 mg/mL fatty acid-free human albumin (FAFA) were seeded in the upper chamber. LPA and inhibitors were prepared in the same migration buffer and placed in the lower chamber. Cells were allowed to migrate for 18 h. Transwell invasion assays were carried out using Corning Biocoat Matrigel Invasion chambers (BD Biosciences, CA, USA) and the cells allowed to invade for 48 h. Non-migrated or non-invaded cells were removed using cotton buds and migrated/invaded cells were rinsed with phosphate buffered saline (PBS) and stained with 0.1% crystal violet (in 20% methanol) for 2.5 h and counted in five random fields for each insert under 20x magnification.

### Organotypic invasion assays

Organotypic cultures with an air–tissue interface were prepared, as described previously (Nystrom et al., 2005; Yap et al., 2009). Briefly, gels comprising a 50:50 mixture of Matrigel (Becton-Dickinson, Oxford, UK) and type I collagen (Upstate) containing 1% (v/v) FBS, 1% (v/v) Hank’s Balanced Salt Solution (HBSS) and 1 × 10^6^/ml ICRF 23 fibroblasts were prepared in cell culture inserts (pore size 3 µm) placed in six-well biocoat plates (Becton Dickinson). After 1 h, sterile glass rings were placed on the gels, and 45 min later DMEM, containing 20% (v/v) foetal clone III (Hyclone), l-glutamine 0.3 µg/ml (FCM) was added to the wells to the level of the insert bases and inside the glass rings. After a further 20 h, the FCM in the glass rings was replaced by 1 ml FAD medium [DMEM: Hams F12 (3:1), 5% FBS(v/v), 5 µg/ml insulin, 10 ng/ml hEGF, 24 ng/ml adenine, 0.4 µg/ml hydrocortisone, 100 U/ml penicillin, 10 µg/ml streptomycin, 250 ng/ml amphotericin B] containing 5 × 10^5^ H357 cells. After a further 24 h, the rings and the media were removed and the media in the wells replaced with rFAD [DMEM:Hams F12 (3:1), 10% FBS, 50 µg/ml l-ascorbic acid, 100 U/ml penicillin, 10 µg/ml streptomycin, 250 ng/ml amphotericin B] supplemented with 5 µM LPA. Media were changed every 48 h, supplemented with 5µM LPA for experimental wells and ethanol:water (1:1 v/v) for controls. After 10 days, the gels were fixed in formal-saline, embedded in a solution containing 1% (v/v) agarose, 10% (v/v) formol saline and processed to paraffin. Sections (2 µm) were stained with H&E.

### Radiation treatment and clonogenic cell survival assays

OSCC cells were seeded at a density of 200,000 cells per dish in 60-mm dishes and cultured overnight before pretreatment with 10 µM LPA for one hour prior to irradiation. In experiments using inhibitors, cells were pretreated with 10 µM LPA for one hour prior to the addition of 10 µM NS-398 for a further one hour before irradiation. Media were changed prior to irradiating the cells with 0-8 gray (Gy) gamma using the Gammacell 3000 (Best Theratronics, Ottawa, Canada). Irradiated cells were harvested immediately by trypsinisation. Cells were counted and 300-500 cells were plated in triplicate for clonogenic survival assays. The cells were incubated for 12 days with media changed every four days and colonies stained with 0.1% (w/v) crystal violet. Colonies with >50 cells were counted as one colony. The plating efficiency (PE) and survival fraction (SF) were calculated using the following equations:

\begin{eqnarray*}\mathrm{PE}= \frac{\text{number of colonies formed}}{\text{number of cells seeded}} \times 100\text{%} \end{eqnarray*}

\begin{eqnarray*}\mathrm{SF}= \frac{\text{PE after irradiation}}{\text{PE of control}} \times 100\text{%} \end{eqnarray*}

### Quantitative real-time polymerase chain reaction (RT-qPCR)

Total RNA extraction and RT-qPCR were carried out as described previously (Lai et al., 2019) The TaqMan^®^ Gene Expression Assays used were PTGS2(Hs00153133_m1) and GAPDH (4326317E) (Applied Biosystems, California, USA). The using the comparative threshold cycle method (ΔΔCt) was used to measure fold changes in gene expression.

### Western blotting

Western blotting was performed as described previously (Lai et al., 2019) The primary antibodies used in this study were anti-COX-2 (SC-1745; 1:500; Santa Cruz Biotechnology, Dallas, USA). and anti-ß-actin (A5441; 1:5000; Sigma Aldrich, St. Loius, USA). Bound antibodies were detected with peroxidase conjugated secondary antibodies and enhanced chemiluminescence reagents (Advansta, Menlo Park, CA, USA). ImageJ was used to measure signal intensities.

### Statistical analyses

All statistical analyses were performed using GraphPad Prism Version 5.01 and SPSS Version 20. Student’s T test, One-way ANOVA with post hoc Dunnett’s test or post hoc Tukey’s test were performed for all experiments.

## Results

### Expression of ENPP2 in normal oral and OSCC tissues

**Figure 1:**
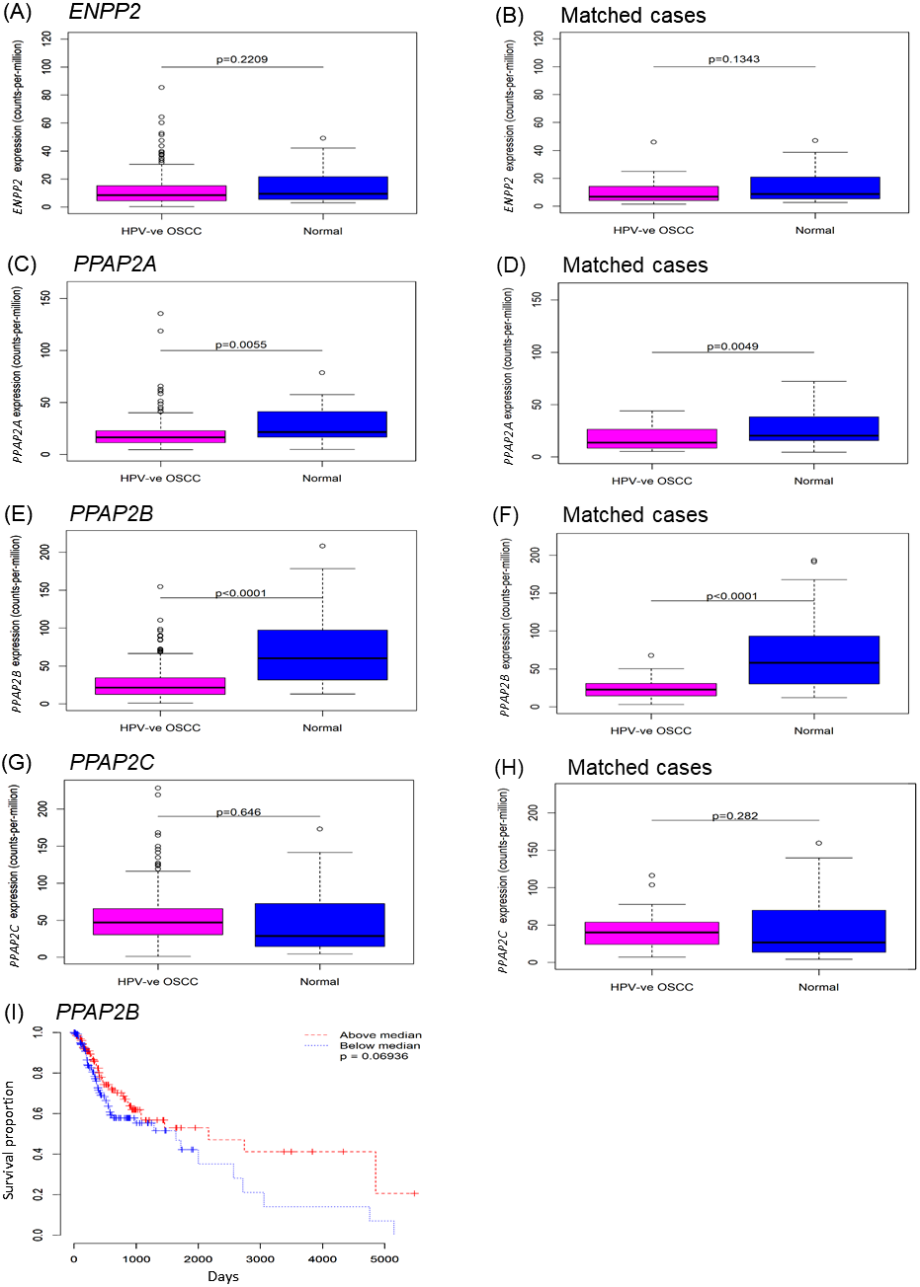
Expression of *ENPP2* and *PPAP2A*-C in normal and OSCC tissues. The expression of genes that encode ATX (*ENPP2*; A, B), LPP1 (*PPAP2A*; C, D), LPP2 (*PPAP2C*; E, F) and LPP3 (*PPAP2B*; G, H) was analysed using expression data from The Cancer Genome Atlas (TCGA). Gene expression was examined in samples of HPV-negative OSCCs and control normal normal mucosa. Where available, gene expression was also examined in OSCCs and patient-matched normal control tissues (B, D, F, H). The expression of *ENPP2A* and *PPAP2C* was not statistically different between tumours and control tissues, whereas the levels of *PPAP2A* and *PPAP2B* were significantly lower in OSCCs than normal mucosa and patient-matched control tissues. Kaplan-Meier plots of overall survival for OSCCs (I) split by median *PPAP2B* expression (log-rank *p* = 0.069).

To investigate ENPP2 and LPP expression in OSCC tissues, we used expression data from The Cancer Genome Atlas (TCGA). The vast majority of OSCCs are not associated with HPV, so we confined our analyses to HPV-negative tumours only, for which data was available for 265 cases. There was no difference in the expression of *ENPP2* in OSCCs relative to normal samples, although it was evident from the box-plots that a small number of cases expressed very high levels of *ENPP2* (Figs. 1A and 1B). By contrast, both LPP1 (*PPAP2A*) and LPP3 (*PPAP2B*) were significantly down-regulated in OSCC tissues, but the levels of LPP2 (*PPAP2C*) were unaltered (Figs. 1C– 1H). We also used the TCGA data to test whether there were any potential associations between expression levels and overall survival in the HPV-negative OSCC cases. For *ENPP2* and *PPAP2A* we observed no such association, but for *PPAP2B* there was some evidence that lower expression may be associated with reduced overall survival, although this was not quite statistically significant. A Kaplan–Meier plot with cases split by median *PPAP2B* expression (log-rank *p* = 0.069) is shown in Fig. 1I. We also performed a Cox proportional hazards analysis using *PPAP2B* expression as the explanatory variable, and this produced a consistent result (Wald *p* = 0.059).

Taken together, these data indicate that a subset of OSCCs expressed elevated levels of *ENPP2* compared to normal oral mucosa, which would lead to the increased production of LPA; the low levels of LPP1 and LPP3 in tumors would augment the effects of LPA (Tang & Brindley, 2020).

### LPA promotes the migration and invasion of OSCC cells

**Figure 2:**
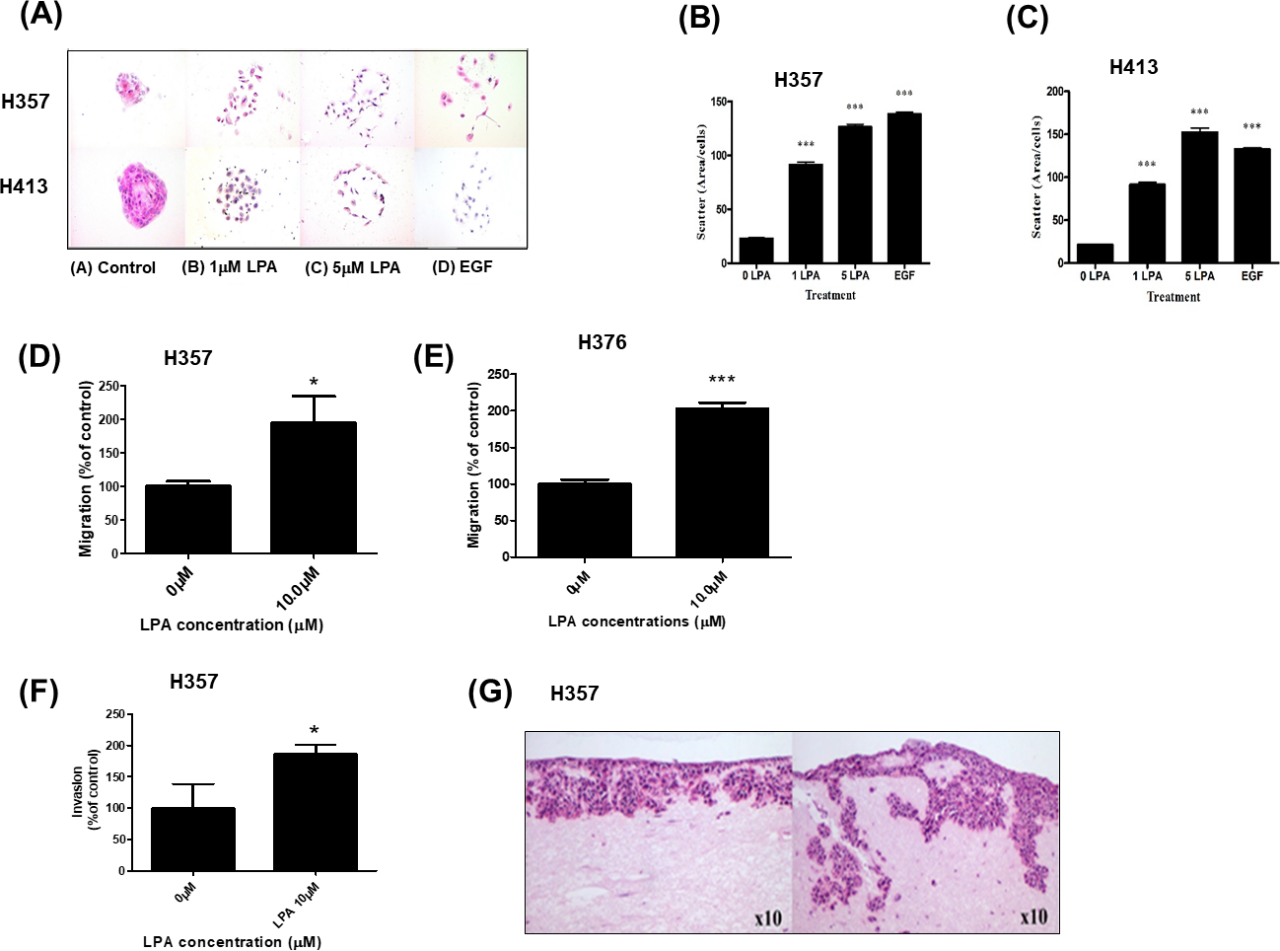
Effect of LPA on OSCC cell migration and invasion. Scatter assays were performed to analyse the effect of LPA on motility of OSCC cells (A). Mitomycin C-treated colonies of H357 and H413 cells in serum-free media were either left untreated of treated with LPA (1 and 5 mM) for 48 h. Epithelial growth factor (EGF; 5 µM) was used as a positive control of cell scatter. Representative images from three independent experiments are shown. (B) The extent of cell scattering for H357 (B) and H413 (C) was analysed by measuring the density of colonies (cells/µm^2^). The data represents the average of measurements taken from thirty colonies chosen at random. Migration of H357 (D) and H376 (E) cells was measured using transwell assays in the absence or presence of 10 µM LPA. Results are expressed as percentage of migrated cells in untreated control (=100%) ± SD. Invasion of H357 cells was measured using Matrigel invasion chambers in the absence or presence of 10 µM LPA (F). Results are expressed as percentage of invaded cells in untreated control (=100%) ± SD. Invasion of H357 cells in organotypic cultures following treatment with LPA or vehicle control for 10 days (G). Images x10 magnification. * indicates *p* < 0.05,^∗∗∗^*p* < 0.001.

As we showed that LPA metabolism was deregulated in OSCCs, we next examined the phenotypic effects of LPA on OSCC cell behaviour in vitro. Scatter assays were performed to assess the effect of LPA on the motility of two OSCC cell lines, H357 and H413. Without treatment, OSCC cells grew to form tightly clustered colonies (Fig. 2A). LPA induced the dispersal of single cells away from the pre-existing colony (Fig. 2A). Quantification of these effects revealed a highly significant increase in motility in response LPA (*P* < 0.001, Figs. 2B and 2C).

We next examined the effect of LPA on OSCC cell migration. LPA significantly enhanced the migration of H357 and H376 cells in transwell assays (Figs. 2D and 2E). H357 cells are able to invade through matrigel in vitro (Patmanathan et al., 2016), so we used this cell line to examine LPA-induced invasion and showed that LPA significantly promoted the invasion of H357 cells through matrigel in transwells (Fig. 2F). Further, in 3D organotypic cultures that are biochemically and physiologically more similar to in vivo tissues than transwells, LPA greatly stimulated the invasion of H357 cells with large clusters of cells spreading deep into the matrix (Fig. 2G).

### LPA protected OSCC cells from the effects of radiation

**Figure 3:**
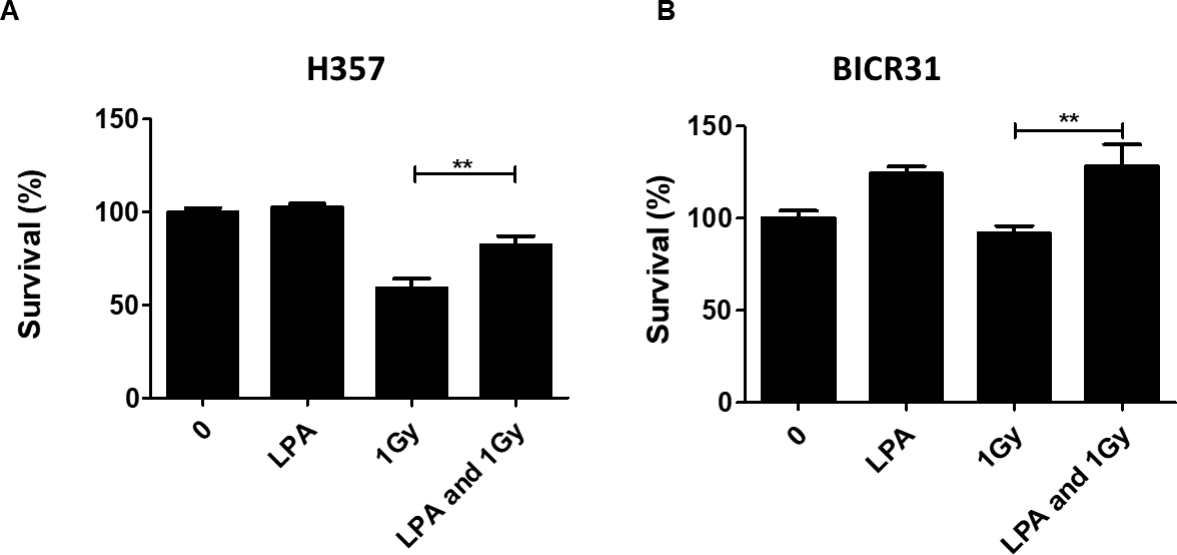
LPA protects OSCC cells against irradiation. (A) H357 or (B) BICR31 cells were pre-treated with LPA (10 µM) for one hour prior to irradiation (1 Gy). Results are expressed as percentage of cells surviving compared to untreated controls (100%) ± SD. Data are representative of three independent experiments. ** indicates *p* < 0.01.

LPA has been reported to suppress apoptosis in non-transformed intestinal crypt-derived epithelial cell lines after high dose radiotherapy (>10Gy) (Deng et al., 2002). Therefore, we investigated the protective effect of LPA on OSCC cells following exposure to radiation using clonogenic cell survival assays. Pre-treatment of cells with LPA increased the survival fractions of both H357 and BICR31 significantly following exposure to 1 Gy irradiation (Figs. 3A and 3B).

### LPA mediates its effects via induction of COX-2 expression

**Figure 4:**
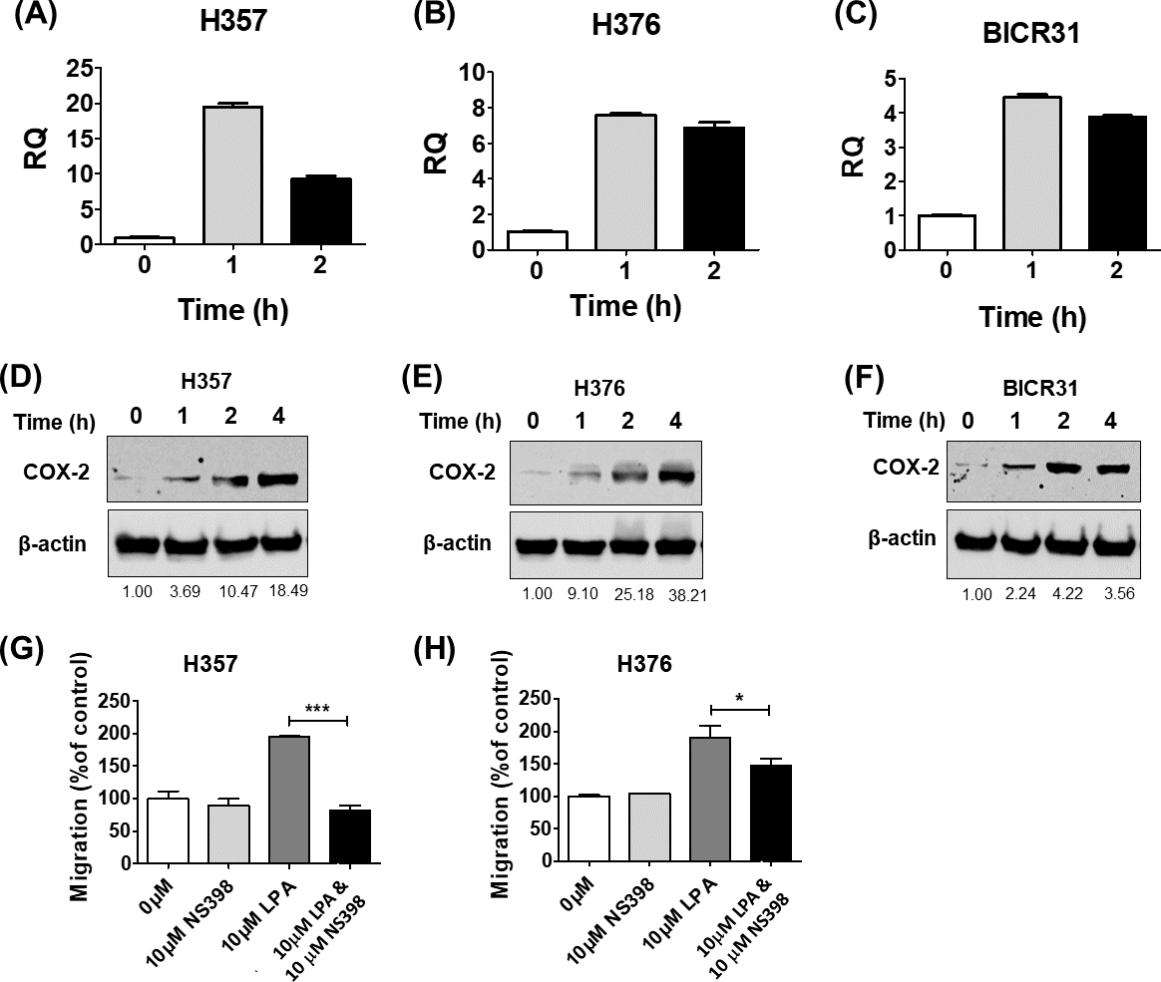
The effects of LPA on OSCC migration are mediated through COX-2. LPA enhanced the transcription of the COX-2 gene (PTGS2). OSCC cells were treated with 10 µM LPA for 1 or 2 h and PTGS2 mRNA levels in H357 (A), H376 (B) and BICR31 (C) determined by RT-qPCR. LPA also induced COX-2 protein expression. 24-hour serum starved H357 (D), H376 (E) and BICR31 (F) cells were treated with 10 µM LPA in serum-free media for the indicated times. Protein lysates were prepared and subjected to immunoblotting to assess COX-2 and ß-actin protein expression. Blots presented are representative of three independent experiments. Densitometric values are shown below the blots and were normalised to ß-actin and expressed relative to time zero (=1). Inhibition of COX-2 attenuated LPA-induced cell migration. Mitomycin-C treated H357 (G) and H376 (H) cells were treated with either vehicle control (50% ethanol), 10 µM LPA, 10 µM NS-398 or a combination of both LPA and NS-398 for 18 h in transwell migration assays. * indicates *p* < 0.05,^∗∗∗^*p* < 0.001.

COX-2 is a known downstream target of LPA signalling and has been shown to regulate cell migration (Jeong et al., 2008) and response to radiation (Terakado et al., 2004). Therefore, we next examined whether LPA could upregulate COX-2 expression in OSCC cells and investigated whether COX-2 mediated some of the phenotypic effects of LPA. Treatment of three OSCC cell lines (H357, H376 and BICR31) with 10 µM LPA resulted in a clear increase in COX-2 mRNA (Figs. 4A– 4C) and protein (Figs. 4D– 4F). To investigate whether the induction of COX-2 expression mediated the effects of LPA on OSCC cell migration and response to radiation, we used a widely used COX-2 inhibitor, NS398, to suppress COX-2 activity. LPA-induced migration of H357 and H376 cells was significantly attenuated following COX-2 inhibition with NS398 in transwell migration assays (Figs. 4G and 4H). NS398 did not affect the survival of LPA-treated H357 and BICR 31 cells exposed to 1 Gy irradiation (Fig. S1).

## Discussion

The prognosis for patients with OSCC is poor with a 5-year mortality rate of around 50%. Therefore, new ways to manage the disease are needed and this is likely to come from the identification of new therapeutic targets. ATX, which is encoded by the ENPP2 gene, is responsible for the generation of LPA from LPC and has been shown to be overexpressed in a number of tumour types, such as melanoma (Sun et al., 2016) and cancers of the thyroid (Benesch et al., 2015), breast (Yang et al., 2002), pancreas (Nakai et al., 2011) and stomach (Zeng et al., 2017), leading to increased levels of LPA in tumours (Nakai et al., 2011; Zeng et al., 2017). In the present study, we show that LPA metabolism is de-regulated in OSCCs. Specifically, we show that whilst only a small proportion of OSCCs expressed high levels of ENPP2, the expression of both LPP1 (PPAP2A) and LPP3 (PPAP2B) was significantly down-regulated in OSCC tissues compared normal controls. These data are consistent with reports that LPP1 and LPP3 are down-regulated in other tumour types (Bhattacharjee et al., 2001; Tang, McMullen & Brindley, 2019; Yoshihara et al., 2009). OSCCs represent a subset of head and neck cancer (HNC) and it has been reported recently that LPP3 is down-regulated in HNCs (Tang & Brindley, 2020). By contrast, LPP2 is often up-regulated in cancers, including HNC , but we did not observe any alterations in LPP2 (PPAP2C) expression specifically in OSCCs. LPP1 and LPP3 play anti-cancer roles and restoration of expression in tumour cells results in suppression of tumour growth, migration and metastasis in mouse models (Tang et al., 2014; Tang, McMullen & Brindley, 2019; Tanyi et al., 2003a; Tanyi et al., 2003b). The anti-tumorigenic roles of LPP1 and LPP3 are thought to be mediated by the breakdown of LPA and also the intracellular activities of the LPPs (Tang & Brindley, 2020). In conclusion, our results indicate that LPA metabolism is deregulated in OSSC leading to elevated levels of LPA and enhanced signalling.

As we showed that LPA metabolism in OSCCs was deregulated, we next examined the phenotypic effects of LPA in OSCC-derived cell lines in vitro. We showed that LPA enhanced the migration and invasion of OSCC cells in a variety of in vitro assays, including 3D organotypic assays of invasion. These results are consistent with previous reports in other tumour types (Hwang et al., 2016; Jourquin et al., 2006; Ren et al., 2019), including OSCC (Brusevold et al., 2014). The effect of LPA on the response of OSCC cells to irradiation was also examined in the present study using clonogenic survival assays and we showed that cells pre-treated with LPA were more resistant to irradiation. Collectively, our data demonstrate that LPA promotes a more migratory/invasive and radio-resistant phenotype in OSCC, data that are consistent with a large body of work describing the tumour promoting effects of LPA in a number of different tumour types (Tigyi et al., 2019).

The LPA signalling pathway is complex with multiple signalling pathways being stimulated downstream of the G protein-coupled LPA receptors. Previously, LPA has been shown to induce COX-2 expression in human colon and ovarian carcinoma cells (Oyesanya et al., 2008; Shida et al., 2005). Similarly, in the present study, we showed that LPA induces COX-2 mRNA and protein expression in OSCC cell lines. Further, we show that LPA-induced migration was attenuated following the inhibition of COX-2 with the widely used COX-2 inhibitor, NS398. To date, COX-2-mediated LPA-induced migration has only been reported in two studies using colon and ovarian carcinoma cells (Jeong et al., 2008; Symowicz et al., 2005). Our data show similar findings in OSCC, observations that not only contribute to a better understanding of OSCC pathogenesis, but also indicate that COX-2 signalling might drive the malignant phenotype in tumours with deregulated LPA metabolism. Further studies are required to dissect the precise LPARs that are responsible for the induction of COX-2 expression and stimulation of more migratory phenotypes in OSCC cells. It has been shown recently, for example, that LPA induces *β*6 integrin expression via LPAR1 in OSCC cells (Xu et al., 2019) and it is noteworthy that COX-2 mediates *α*v*β*6 integrin-dependent invasion in OSCC (Nystrom et al., 2006).

## Conclusions

In conclusion, we show that LPA metabolism is deregulated in OSCCs by the down-regulation of LPP1 and LPP3 and this contributes to a more migratory phenotype in OSCC. We further show that the pro-migratory effects of LPA are mediated via the induction of COX-2 expression. Our results highlight the potential of targeting LPA metabolism alongside the inhibition of COX-2 in the clinical setting.

##  Supplemental Information

10.7717/peerj.10328/supp-1 Supplemental Information 1 NS398 did not reverse the effects of LPA in irradiated OSCC cells (A) H357 or (B) BICR31 cells were pre-treated with LPA (10µM) for one hour prior to treatment with 10 µM NS-398 for a further one hour prior to irradiation (1 Gy). Results are expressed as percentage of cells surviving compared to untreated controls (100%) .

10.7717/peerj.10328/supp-2 Supplemental Information 2 Raw data for Figs. 2–4

## Additional Information and Declarations

### Data Availability

The following information was supplied regarding data availability: The raw data for Figs. 2–4 are available as a Supplemental File.
