# Supplementary material for: Deregulation of lysophosphatidic acid metabolism in oral cancer promotes cell migration via the up-regulation of COX-2
Source: PeerJ. 2020 Nov 11;8:e10328. doi: 10.7717/peerj.10328 (PMC7666559; doi:10.7717/peerj.10328)
Supplement: Supplemental Information 1 — (A) H357 or (B) BICR31 cells were pre-treated with LPA (10µM) for one hour prior to treatment with 10 µM NS-398 for a further one hour prior to irradiation (1 Gy). Results are expressed as percentage of cells surviving compared to untreated controls (100%) . [file peerj-08-10328-s001.pdf]

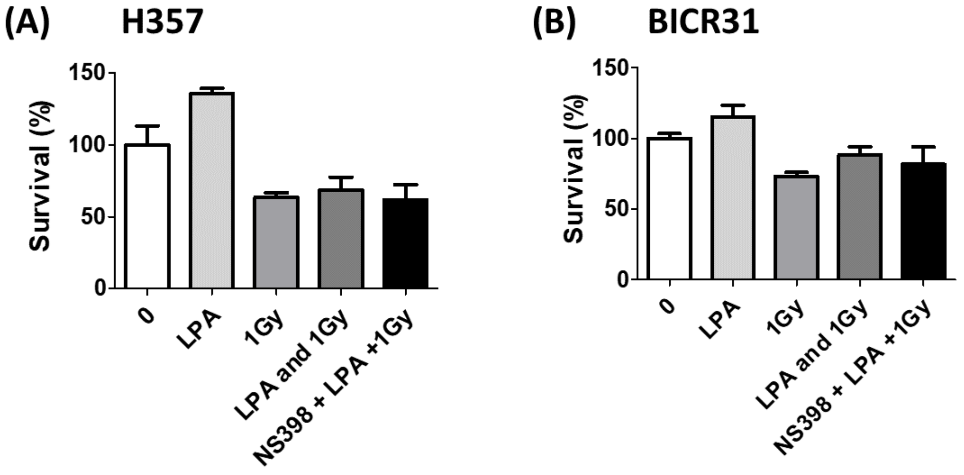

Figure S1. NS398 did not reverse the effects of LPA in irradiated OSCC cells
(A) H357 or (B) BICR31 cells were pre-treated with LPA (10µM) for one hour prior to treatment with 10 µM NS-398 for a further one hour prior to irradiation (1 Gy). Results are expressed as percentage of cells surviving compared to untreated controls (100%) .
